# Bacterial meningitis as a first presentation of pituitary macroprolactinoma

**DOI:** 10.1530/EDM-14-0028

**Published:** 2014-05-01

**Authors:** Niki Margari, Simon Page

**Affiliations:** 1 HaematologyLincoln County HospitalLincolnUK; 2 Nottingham University Hospitals NHS TrustNottinghamUK

## Abstract

A 56-year-old man was brought to the Emergency Department after being found collapsed at his office with a reduced level of consciousness. From clinical examination and initial investigations, he was diagnosed as having bacterial meningitis and was promptly commenced on empirical i.v. antibiotics. Computed tomography of the brain revealed a parenchymal mass at the base of the skull and subsequent magnetic resonance imaging of the head 4 days later confirmed a large soft tissue mass, which extended through to the cavernous sinus. Examination of the cerebrospinal fluid (CSF) following lumbar puncture confirmed pneumococcal meningitis and antibiotics were continued for 2 weeks in total. During the admission, hormone profiling revealed a grossly elevated prolactin. When coupled with the initial results of the brain imaging, this result helped to confirm a macroprolactinoma that was invading the postnasal space. A final diagnosis of pneumococcal meningitis secondary to invading prolactinoma was made. The patient was started on cabergoline and was followed up in the outpatient clinic upon discharge. He made a full recovery from the meningitis. Over the next few months, prolactin levels returned to be normal and the prolactinoma shrank significantly in size. The patient remains on cabergoline that will most likely be continued indefinitely.

**Learning points:**

- Bacterial meningitis is a rare first presentation of pituitary macroprolactinoma.
- Patients with invasive macroprolactinoma do not always present with CSF leakage.
- Prompt treatment with antibiotics and a dopamine agonist is of great importance for a favourable outcome.
- Close monitoring of the patient for signs of raised intracranial pressure is essential in the management of macroprolactinoma.
- Note the risk of CSF leakage after initiation of dopamine agonist therapy irrespective of concomitant meningitis in macroprolactinoma.

## Background

Bacterial meningitis as a first presentation of pituitary macroprolactinoma is very rare. This case highlights the importance of careful clinical assessment and focused investigations, including cranial imaging and pituitary hormone profiling to establish the underlying diagnosis. Dopamine agonists are the treatment of choice for invasive macroprolactinoma and close monitoring is essential as there is a risk of cerebrospinal fluid (CSF) leakage as the tumour undergoes involution.

## Case presentation

A 56-year-old man with no past medical history and not on any regular medication was brought to the Emergency Department by the paramedics after he was found collapsed at work by colleagues. Collateral history from family and friends excluded any prodromal symptoms before collapse. On admission, his Glasgow Coma Scale was 9/15 (eyes 3, motor 5 and verbal 1). Aside from being febrile with a temperature of 38.7 °C, the remaining vital signs were otherwise normal and stable. On examination, there was no focal neurological deficit, but marked neck stiffness was elicited.

## Investigation

**Figure 1:**
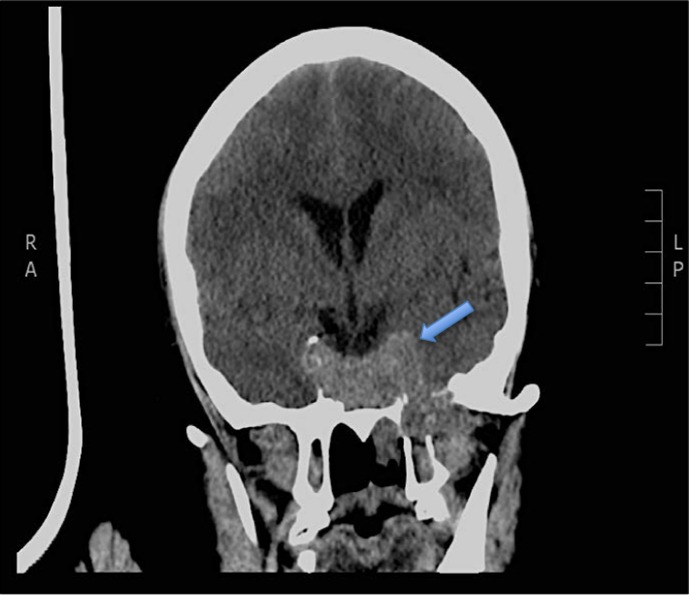
Computed tomography brain scan that revealed a parenchymal mass at the base of the skull eroding the body of the clivus (blue arrow).

Raised inflammatory markers and clinical presentation prompted an urgent computed tomography head scan on the day of admission that revealed a parenchymal mass at the base of the skull eroding the body of the clivus (Fig. 1). A subsequent lumbar puncture demonstrated markedly raised protein in the CSF (9523 g/l) with very low glucose (<1.1 mmol/l) and polymorphs of 2240 (normal 0). Pneumococcal polymerase chain reaction later came back positive. In addition, urine pneumococcal antigen was positive, which helped to confirm the diagnosis of pneumococcal meningitis.

**Figure 2:**
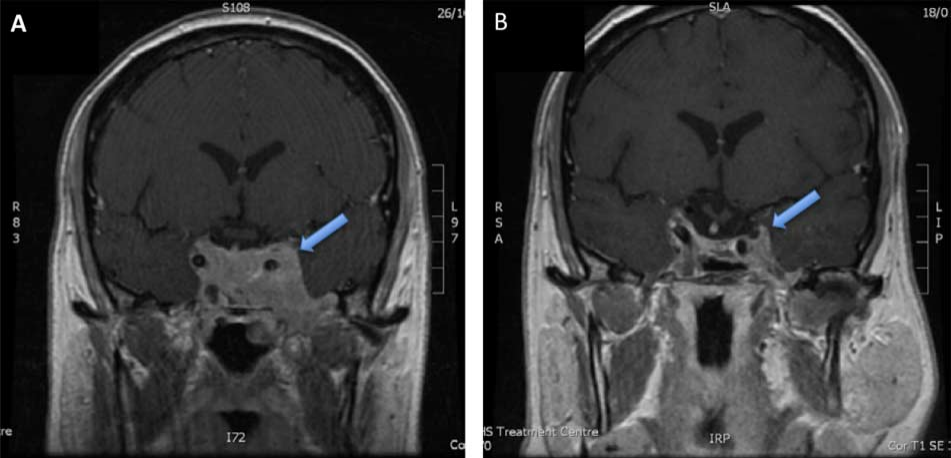
Cranial magnetic resonance imaging scan revealing a large soft tissue mass at the base of the skull (blue arrow, plate A). Following treatment with cabergoline, the soft tissue mass reduced in size significantly after 3 months of therapy (blue arrow, plate B).

The patient underwent cranial magnetic resonance imaging (MRI) four days later for enhanced visualisation of the tumour, which delineated a soft tissue mass invading the left cavernous sinus (Fig. 2A). Neurosurgical advice was sought. It was thought that biopsy of the mass was appropriate for tissue diagnosis following discussion between the neurosurgeons and radiologists to exclude possible squamous cell carcinoma or brain abscess. Surgical intervention was not indicated at that juncture, as the patient did not have any symptoms arising from compression of surrounding structures. Visual fields were normal. The patient was closely monitored for signs of raised intracranial pressure and evidence of CSF leakage (e.g. rhinorrhoea or otorrhoea), neither of which ultimately developed. No further imaging was performed.

During the admission, the endocrinology team were also involved. Inpatient measurement of pituitary hormones revealed: thyroid-stimulating hormone 2.9 mIU/l with free T_4_ 13.9 pmol/l and free T_3_ 3.8 pmol/l, 0900 h cortisol >2069 nmol/l, luteinizing hormone 0.9 IU/l (1.5–18.1), follicle-stimulating hormone 0.8 IU/l (1.4–18.1), random growth hormone 0.10 ng/ml, insulin-like growth factor 1 101 ng/ml (which is normal), testosterone 2.6 nmol/l (normal ≥6.2) and a markedly raised prolactin 133 100 mIU/l. Given the results of the brain imaging alongside the pituitary hormone profiling, a diagnosis of bacterial meningitis secondary to invading prolactinoma was made.

## Treatment

The patient was commenced on i.v. ceftriaxone 2 g once daily and amoxicillin 2 g every 4 h for 2 weeks to treat the pneumococcal meningitis.

The hormone profiling demonstrated abnormal pituitary function along with suppression of gonadotrophin and testosterone levels. As such, the patient was started on cabergoline 500 μg twice weekly. The patient was observed on the ward for signs of CSF leakage as the tumour underwent involution but none were noted. The patient had not complained of loss of libido or tiredness despite the low gonadotrophin and testosterone levels.

## Outcome and follow-up

The patient was discharged after a complete and uneventful recovery. He was followed up in the endocrine clinic a week later when his prolactin level had fallen significantly to 15 633 mIU/l. A pituitary multidisciplinary meeting recommended that the patient should be maintained on cabergoline indefinitely with regular endocrine follow-up. He had a repeat MRI head scan 3 months after initiation of the treatment, which showed a significant reduction in the size of the prolactinoma (Fig. 2B). With sustained cabergoline treatment, the patient's prolactin has fallen into the normal range and his pituitary function remains otherwise intact.

Despite the diagnosis and treatment of macroprolactinoma during the admission, the neurosurgeons performed a biopsy of the mass as an elective procedure following discharge once the meningitis had resolved. The biopsy confirmed macroprolactinoma and excluded both squamous cell carcinoma and brain abscess.

## Discussion

This is a rare presentation of a pituitary tumour. Macroprolactinomas more commonly present with clinical features of hyperprolactinaemia, such as loss of libido and impotency in males or galactorrhoea and menstrual dysfunction in females. They can also present as space-occupying lesions with headaches, vomiting and visual disturbances, although our patient did not have any of these symptoms. In the literature, only 52 isolated cases were found with CSF leaking due to invading macroprolactinoma (14 patients) or medically induced CSF leakage (38 patients) and only seven of these went on to develop meningitis (1). The commonest pathogens were *Streptococcus pneumoniae*, *Neisseria meningitidis* and *Haemophilus influenzae*. This case is particularly interesting, as there was no CSF leakage apparent, which would theoretically increase the susceptibility to develop meningitis. Indeed, there have been only two cases reported with prolactinoma and meningitis but no rhinorrhoea as a sign of CSF leakage (2).

Medical treatment with dopaminergic agonists such as bromocriptine and cabergoline is considered as first-line treatment for macroprolactinomas compared with other pituitary tumours in which surgical intervention may be required. The patient better tolerates cabergoline, which is more effective than bromocriptine in reducing the size of the tumour and secretion of prolactin (3). Surgery is reserved for patients who do not respond to medical treatment or when there is significant CSF leaking at which point surgical repair of the dural defect is necessary.

## Patient consent

Written informed consent was obtained from the patient for publication of the submitted article and accompanying images.

## Author contribution statement

N Margari wrote the case report in its entirety. S Page critically revised the paper and both authors have seen and approved the final manuscript for submission.
